# Magmatic karst reveals dynamics of crystallization and differentiation in basaltic magma chambers

**DOI:** 10.1038/s41598-021-86724-y

**Published:** 2021-04-01

**Authors:** Willem Kruger, Rais Latypov

**Affiliations:** School of Geosciences, University of the Witwatersrand, Johannesburg, South Africa

**Keywords:** Geochemistry, Geology, Mineralogy, Petrology, Volcanology

## Abstract

An understanding of magma chamber dynamics relies on answering three important yet highly controversial questions: where, why, and how magma chambers crystallize and differentiate. Here we report on a new natural phenomenon—the undercut-embayed chamber floor in the Bushveld Complex—which allows us to address these questions. The undercut-embayed floor is produced by magmatic karstification (i.e. erosion by dissolution) of the underlying cumulates by replenishing magmas that form basal flows on the chamber floor. This results in a few metres thick three-dimensional framework of spatially interconnected erosional remnants that separate the floor cumulates from the overlying resident melt. The basal flow in this environment is effectively cooled through the floor, inducing heterogeneous nucleation and in situ growth against much of its three-dimensional framework. The solidification front thus propagates in multiple directions from the surfaces of erosional remnants. Fractional crystallization may occur within this environment by convective removal of a compositional boundary layer from in situ growing crystals and is remarkably efficient even in very confined spaces. We propose that the way magma crystallizes and differentiates in the undercut-embayed chamber floor is likely common for the evolution of many basaltic magma chambers.

## Introduction

Gaining deeper insights into how natural magmas crystallize and differentiate in crustal magma chambers is crucial for scientific investigations in many fields of igneous petrology, from highly detailed studies at the scale of individual crystals^1,2^ to global processes concerning the evolution of Earth and other terrestrial planets^3–5^. A complete understanding of the inner workings of magma chambers relies on answering three fundamental questions: Where do crystallization and differentiation occur? Why do crystallization and differentiation occur within this particular environment? What processes are responsible for crystallization and differentiation? A plethora of models exist on each of these questions. For instance, in regard to the first question, models may portray magma chambers as melt pools in which crystals either grow on pre-existing crystals along the chamber margins^6–9^ in structures called solidification fronts^10–13^, form within the melt and then settle on the chamber floor^14–19^, or are kept suspended in a convecting melt until the formation of crystal-rich mush that is unable to flow^20–22^. A more recently proposed model involves the crystallization and transport of crystals in an interconnected, transcrustal system of dykes and sills^23^. These crystals may eventually be deposited in a magma chamber when the melt becomes unable to carry the crystal load to form a layered mafic intrusion^24^. Critical information necessary to resolve these and many other contrasting interpretations is difficult to obtain because evolving magma chambers are hidden from our direct observation.

One way to address this problem is to examine the solidified remains of mafic–ultramafic intrusions—fossilized natural laboratories that constrain many fundamental principles of igneous petrology^17,25,26^. Here we report on intricate chemical patterns in massive magnetitites of the Bushveld Complex in South Africa^27^ that enables the recognition of a new petrological phenomenon in magma chambers—the undercutting and embayment of the temporary chamber floor. The ‘undercut-embayed floor’ is unique in providing definitive constraints on magma crystallization and differentiation processes, thereby providing explicit solutions to the above fundamental questions.

## Chemical patterns in massive magnetitite

**Figure 1:**
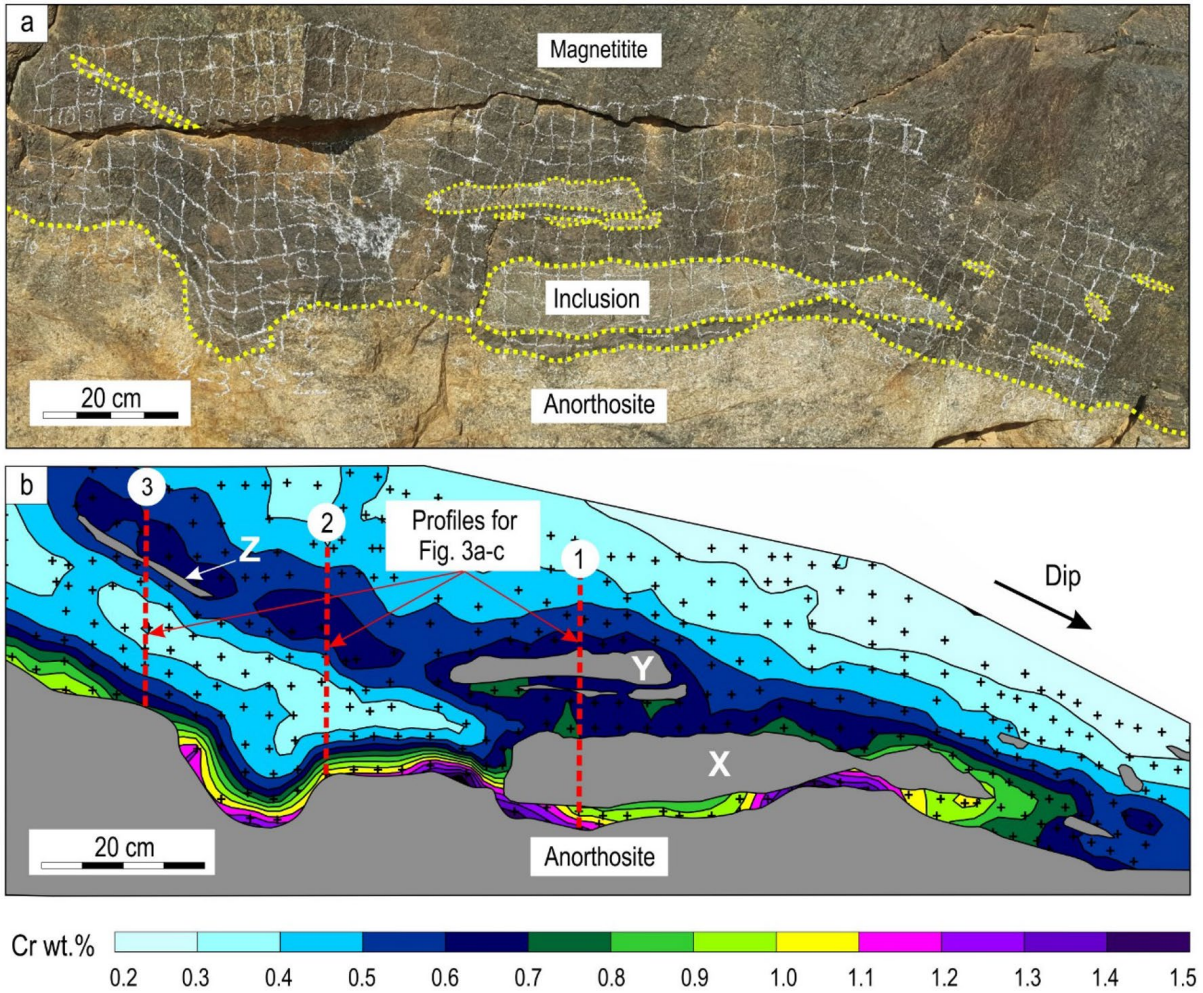
Massive magnetitite with two-dimensional Cr chemical patterns. **(a)** Field photograph of an outcrop of the bottom seam of massive magnetitite showing undulated footwall contact and several anorthositic inclusions; **(b)** Geochemical contour map showing the distribution of Cr within the layer. Crosses indicate individual analysis points. Overall, the Cr concentration decreases rapidly upwards in the layer. Higher Cr contents were recorded at the basal contact and around anorthositic inclusions, including a cryptic zone with higher Cr contents connecting the central inclusion Y and a smaller one Z to the left. A converging chemical pattern is observed between this cryptic zone and the floor. Several dome-shaped high Cr growth nodes on the bottom contact of the layer indicate incipient in situ nucleation and growth of magnetitite^35,52^. Two-dimensional geochemical mapping of this outcrop is done using a handheld X-ray fluorescence spectrometer (pXRF). Black crosses indicate the positions of individual data points. The pXRF data can be found in Supplementary Information Table 1. Figure 1b is created using Surfer software (version 9.2.397). The outcrop is located at the Rhovan Mine, Western Bushveld Complex, South Africa.

**Figure 2:**
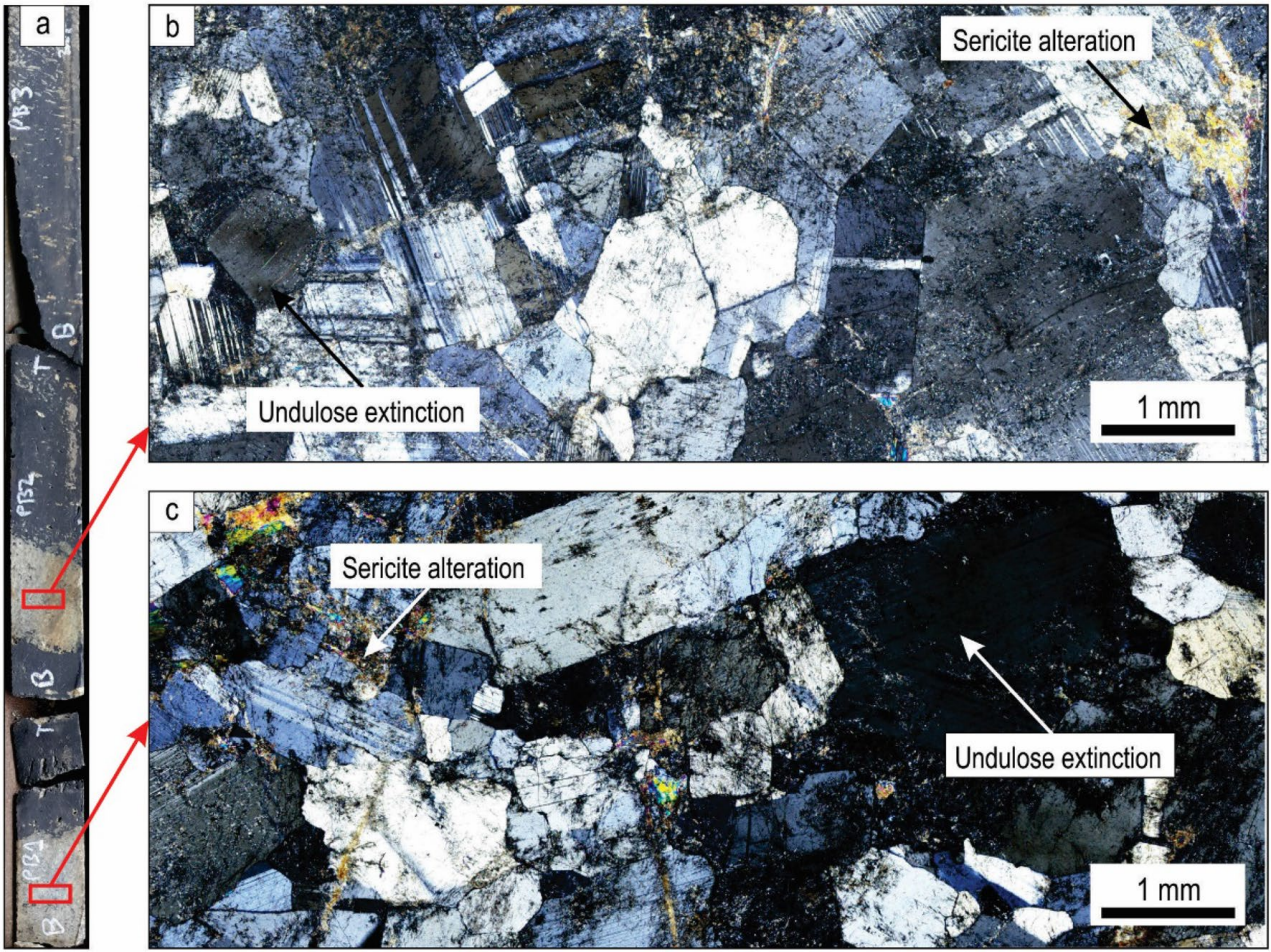
Petrography of footwall anorthosite and anorthosite inclusions in massive magnetitite. **(a)** Drill core from which samples were collected. The drill core measures about 4.5 cm across. Thin sections of both an inclusion **(b)** and the footwall anorthosite **(c)** are shown in crossed polarized light. The two samples are petrographically indistinguishable from each other and are both characterized by the near-absence of any primocrysts other than plagioclase, a similar degree of deformation in the form of undulose extinction, a seriate fabric and some degree of sericite alteration.

We have examined a spectacular outcrop of the lowermost magnetitite layer (also termed as the bottom seam) in the Bushveld Complex—the largest preserved layered intrusion in Earth’s crust^27^. This layer shows peculiar field relationships with the underlying anorthosite (Fig. 1a) such as depressions and undulations, while the most striking feature is several elongated anorthositic inclusions trapped within the magnetitite. From a textural perspective the inclusions are identical to the footwall anorthosite (Fig. 2). Such inclusions within massive magnetitite have previously been interpreted as “partially resorbed xenoliths”^28^, although no further details on the nature of the inclusions have been provided. In layered intrusions, such inclusions can be viewed as transported fragments that have either been brought into the chamber with inflowing magmas^29^ or fallen from the roof sequence onto the chamber floor^5,30–32^.

They may also be interpreted as in situ remnants of originally continuous layers that were dissolved by thermochemical erosion associated with magma chamber replenishment^33,34^. A commonality for these interpretations is that after mechanical deposition^5,29–32^ or in situ formation^33,34^ the fragments are supposed to lie directly on the chamber floor, with no open space filled with resident melt below them. We have examined this outcrop following a procedure from our recent study of trace element (e.g. Cr) distributions in magnetitites in two dimensions by a handheld X-ray fluorescence spectrometer (pXRF)^35^ (Fig. 1b; Supplementary Information Fig. 1). Special attention has been given to Cr because it is extremely sensitive to record magmatic crystallization and differentiation^35,36^. This is primarily due to the exceptionally high magnetite-liquid partition coefficients in basaltic melts that may range from 290 to beyond 600^36–39^.

In line with previous studies^36^, our two-dimensional mapping of the magnetitite layer reveal a dramatic depletion in Cr contents upwards. To produce such a strong upwards depletion, previous investigators^36,40^ proposed that magnetitite layers of the Bushveld Complex had to crystallize from a limited volume of melt at the base of the magma chamber that does not mix with the magma chamber’s convecting interior. Such a basal melt layer can be rapidly depleted in Cr during the crystallization of massive magnetitite compared to the case where Cr is sourced from the entire magma chamber^35,36,40^. There are many plausible explanations for how such a basal melt layer may form that varies from double diffusive convection^40–42^ to stagnation of melt at the chamber floor^36,43^. In our recent work^35^, we proposed that the layer originated by the introduction of a hot magma pulse into the chamber. This conclusion stems from several lines of evidence that suggests magmatic recharge occurred prior to the formation of magnetitite layers^28,35,41^. Because the incoming magma forms a thick layer of massive magnetitite, it is expected to be richer in iron and, therefore, has a higher density than the resident melt. This causes the incoming melt to spread out across the chamber floor as a basal flow underneath the resident melt during its emplacement^44,45^. The existence of basal flows is evidenced by thermochemical erosion of chamber floor cumulates within the Bushveld Complex on a regional scale^33,34,46–49^.

**Figure 3:**
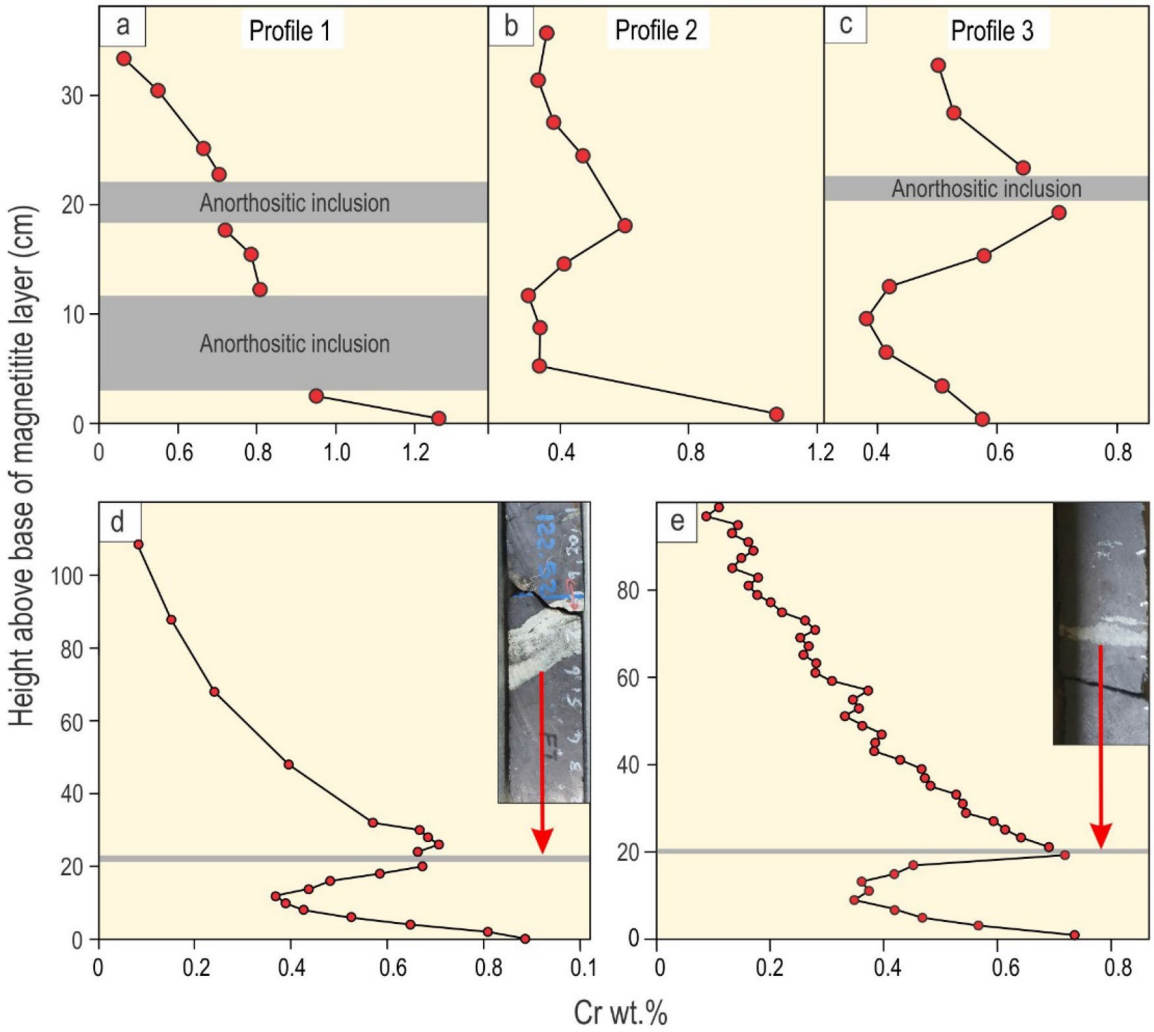
One-dimensional vertical profiles in Cr content across a massive magnetitite. Position of these three profiles across the bottom seam of massive magnetitite is indicated on Fig. 1b. **(a)** A rapid continuous decrease in Cr content upwards with no chemical changes/breaks at anorthositic inclusions; **(b)** A gradual reversal in Cr content in a level where no anorthositic inclusions are visible; **(c)** An initially rapid decrease in Cr content followed by its gradual increase towards an anorthositic inclusion. Cr content again declines upwards from the top of the anorthositic inclusion. **(d)** and **(e)** Additional examples of vertical profiles in Cr content across the bottom seam that contains anorthosite inclusions from drill cores in a different locality (Vametco mine, Western Bushveld Complex). Insets show anorthositic inclusions (light in colour) trapped within the massive magnetitite (dark in colour). The distribution of the Cr in these two examples is similar to that in **(c)**. Vertical geochemical profiles are analysed using a handheld X-ray fluorescence spectrometer (pXRF). The pXRF data for Fig. 3a–c can be found in Supplementary Information Table 1, for Fig. 3 d in Supplementary Information Table 2 and Fig. 3e in Supplementary Information Table 3. Geochemical profiles are prepared using Microsoft Excel 2013 (15.0.5319.1000) and CorelDRAW (version 18.1.0.690).

The general upward decrease in Cr within the magnetitite layer (Fig. 1b) suggests that magnetite was not held in suspension by vigorous convection^20–22^ but rather crystallized directly at the base of the magma chamber to form the cryptic layering patterns. We interpret these patterns as recording the morphology of a solidification front as it grows^10–13^. The study of such chemical patterns provides an unparalleled visualization of step-wise propagation of solidification fronts in a magma chamber^35^. An interesting feature of the geochemical contour map is that Cr contents generally appear elevated in the vicinity of anorthositic fragments. We have also disclosed a cryptic zone of magnetite with an elevated Cr concentration (blue in colour) that connects a small anorthositic slither to the left (Z) and a larger inclusion (Y) towards the centre of the outcrop. In selected one-dimensional profiles, three distinct compositional trends are observed: a continuous upward depletion in Cr across fragments (Fig. 3a), a gradual reversal in Cr where no fragments are present (Fig. 3b), and an inward decrease in Cr away from the footwall and a fragment (Fig. 3c). Two more examples with the inward decrease in Cr through the bottom seam are additionally shown from other areas (Fig. 3d, e).

Before discussing the results any further, it is of importance to determine if the observed chemical patterns are of primary magmatic origin or result from some fluid activity and/or reaction with the anorthositic footwall or inclusions. For example, it was recently proposed that anorthosite layers of the Bushveld Complex represent proto-norite in which mafic minerals were dissolved by a fluid^50^. Dissolving of pyroxene may cause a fluid to become enriched in Cr, leading to the enrichment of magnetite in this trace element in the vicinity of the footwall and anorthositic inclusions. However, gabbro that occurs below the anorthositic footwall of the magnetitite layer has a Cr concentration less than 90 ppm^51^ whereas magnetitite at the base and around inclusions contain Cr in excess of 8000 ppm (Fig. 1b). This process is, therefore, considered extremely unlikely to be responsible for the elevated Cr contents observed in the studied profiles (Figs. 1 and 3).

### The phenomenon of the undercut-embayed floor

In line with some previous studies^35,52^, we have recorded high Cr structures (purple in colour; Fig. 1b) that indicate sites of incipient nucleation of magnetitite at the very base of the profile. This was followed by self-nucleation on the pre-existing nuclei, causing outward concentric growth to produce dome-shaped structures that are referred to as in situ growth nodes. Magnetite growth at the base proceeded until most of the footwall was covered. Shortly thereafter, magnetite must have started nucleating and growing directly on the surfaces of anorthositic inclusions as indicated by the elevated Cr contents of magnetite in their vicinity. Locally, solidification fronts growing from the floor upwards and from the inclusions downwards converged, resulting in cryptic layers that are depleted in Cr relative to the magnetitite directly above and below (Supplementary Information Movie 1). This omnidirectional growth of magnetite around anorthositic inclusions leads to the most intriguing conclusion: these fragments must have been present in their current positions and entirely surrounded by the melt prior to onset of its crystallization. This finding precludes the models that imply that fragments had to be resting directly on a solid floor^5,29–32^. Clearly, these fragments could not remain suspended in the melt near to the floor; they would either settle or float depending on their density relationships with the resident melt. A case where these fragments are neutrally buoyant is considered extremely unlikely. According to Stoke’s Law, even a 1% difference in density compared to the melt can cause such fragments smaller than 10 cm to sink or float several metres a week (assuming a liquid viscosity of 10^4^ Pa.s) (Methods).

In contrast, solidification fronts only propagate at a rate of a few cm per year^53^. To prevent their movement, the inclusions must somehow have been anchored in their current positions. The cryptic zone of magnetite enriched in Cr that connects two inclusions (Z and Y, Fig. 1b) provides an important clue to this issue. The continuous nature of this zone as well as the fact that the reversal appears to be gradual (Figs. 1b and 3b) suggests that magnetite was growing on some septum that is now either hidden behind the current face of the outcrop or has already been removed by mining. This septum is most likely composed of a continuous anorthosite body that interconnects these two and likely many other obscured inclusion-like bodies. All the seemingly separate anorthositic inclusions in this outcrop are thus likely interlinked with each other and firmly attached to the footwall in three dimensions. We refer to this three-dimensional framework of spatially interconnected bodies at the bottom of the chamber as the ‘undercut-embayed floor’.

### Magmatic karstification of the floor cumulates

**Figure 4:**
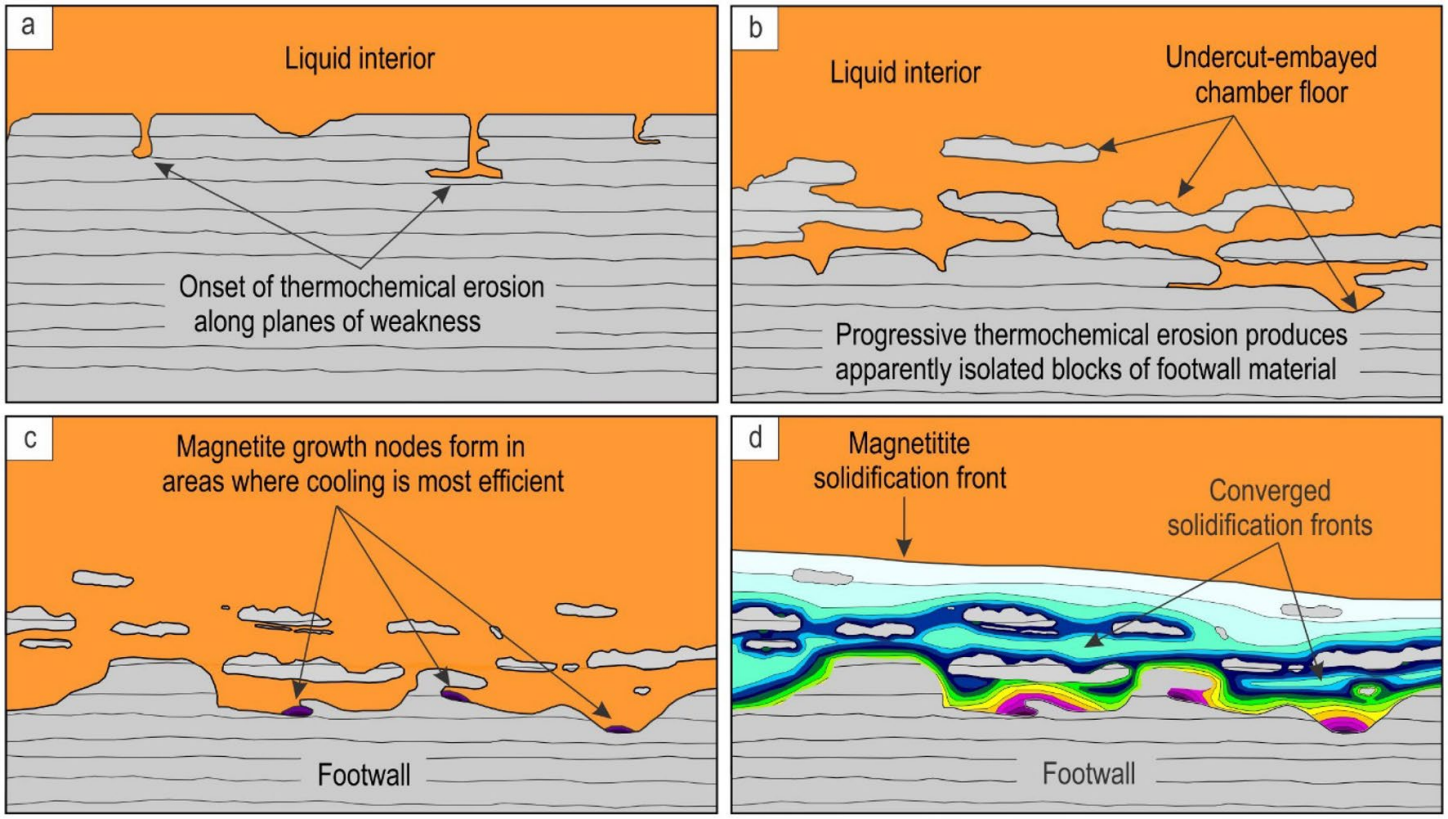
Physical model for the origin of the undercut-embayed chamber floor and propagation of a solidification front therein. **(a)** Floor cumulates with an initial planar surface undergo thermochemical erosion along points/planes of weakness by a newly-emplaced reactive melt; **(b)** As erosion progresses, blocks that appear isolated in two dimensions but still connected with the floor in three dimensions may form, eventually resulting in the undercut-embayed chamber floor. **(c)** With time, thermochemical erosion comes to a halt and, after some degree of cooling, magnetite starts to nucleate and grow in areas where heat loss is most rapid, such as in depressions or underneath fragments partly attached to the floor. **(d)** Crystal nucleation and growth from the floor and fragments results in omnidirectional propagation patterns of the solidification front, with some converging solidification patterns in the vicinity of the chamber floor. The figure is prepared using CorelDRAW (version 18.1.0.690).

**Figure 5:**
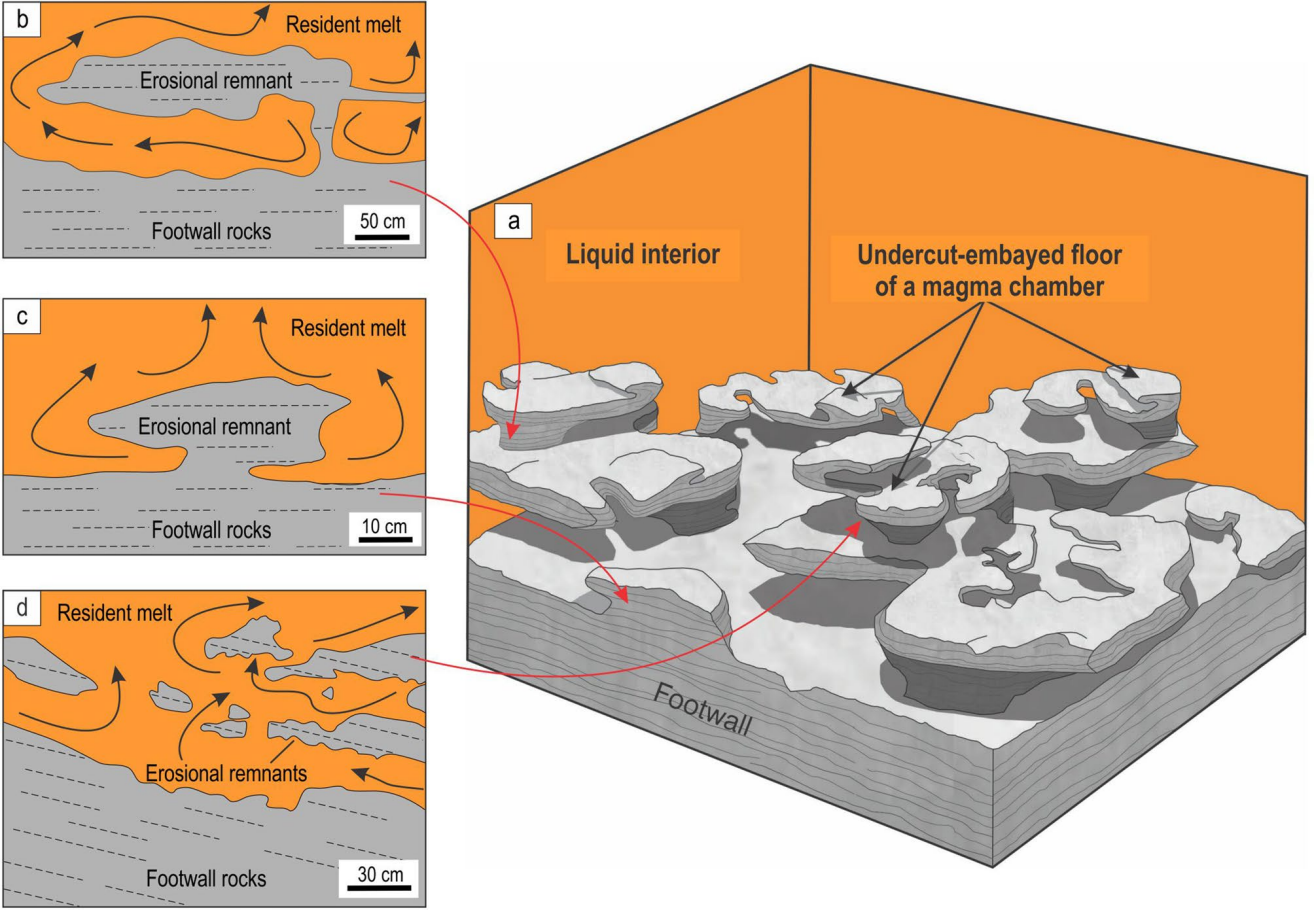
Artistic impression of the undercut-embayed chamber floor in an evolving magma chamber along with interpretive sketches of relevant exposures. **(a)** When the undercut-embayed floor is observed in three dimensions, it becomes obvious that most seemingly isolated ‘inclusions’ in two dimensions are actually connected with each other to produce an intricate in three-dimensional framework of partially eroded floor cumulates. The process responsible for the formation of such a floor is referred to as here as magmatic ‘karstification’. The undercut-embayed floor is expected to be common in open magma chambers. **(b)** Interpretive sketch of an erosional remnant of anorthosite that is hosted by the Main Magnetite Layer and is still attached to the footwall rocks. Vametco Vanadium Mine, Upper Zone of the Western Bushveld Complex (Fig. 6a). **(c)** Interpretive sketch of an erosional remnant of orthopyroxenite that is hosted by the Lower Group 6 (LG6) chromitite and is still attached to the footwall rocks. Jagdlust area, Lower Critical Zone of the Eastern Bushveld Complex (Fig. 6b). **(d)** Interpretive sketch of in situ erosional remnants of anorthosite that are hosted by the Merensky Reef orthopyroxenite and seemingly not attached to the footwall rocks in this section. Rustenburg Platinum Mine, Upper Critical Zone of the Western Bushveld Complex (Fig. 6e). Black arrowed curves in (b–d) show compositional convection. Red arrowed curves indicate possible positions of these 2D exposures in the 3D space of the undercut-embayed chamber floor. The figure is prepared using CorelDRAW (version 18.1.0.690).

The undercut-embayed floor can be best explained as a result of thermochemical erosion of the basal cumulates by highly reactive and likely superheated melts replenishing the evolving Bushveld chamber^34,46,47^. Replenishing melts may possess high erosive power due to their chemical and thermal disequilibrium with the pre-existing floor cumulates. Thermal disequilibrium results from melt superheating that can be up to 90 °C for basaltic melts rising near adiabatically from the deep-seated magmatic reservoir^54^. Even if some cooling of the ascending melt takes place so that it arrives into the chamber at a much lower degree of superheating (say, 10–15 °C), a few tens of metres’ thick column of such a melt can still erode a few metres of footwall rocks^46,47^ (Methods). This is because a major agent of magmatic erosion is not the heat itself (causing melting) but rather chemical disequilibrium (causing dissolution) between the new melts and floor cumulates^54,55^. Melt superheating is still, however, essential in preventing the onset of melt crystallization. The reason is that the formation of a new basal layer of rocks may immediately terminate the dissolution of the floor cumulates^34^. Geochemical evidence suggests that magmatic recharge of the chamber preceded the formation of massive magnetitite layers in the Bushveld Complex^28^. If these new pulses are not in chemical and thermal equilibrium with the floor rocks, the melt may cause their whole-sale thermochemical erosion. With time, the intensity of erosion will wane to become only partial—mostly occurring along fractures and planes of weakness (Figs. 4, 5)—and will result in the complex, undercut-embayed morphology of the floor cumulates. We suggest referring to this phenomenon as magmatic ‘karstification’ of the chamber floor cumulates as it is similar in both morphology and origin to karst landforms in surface sedimentary rocks produced by infiltrating acidic water^56^.

### Challenging the fundamentals of magma chamber dynamics

The chemical patterns in the magnetitite layer (Fig. 1) challenge the universal validity of several fundamental principles of magma chamber dynamics which are deeply entrenched, although not always explicitly formulated, in modern petrological concepts.

**Figure 6:**
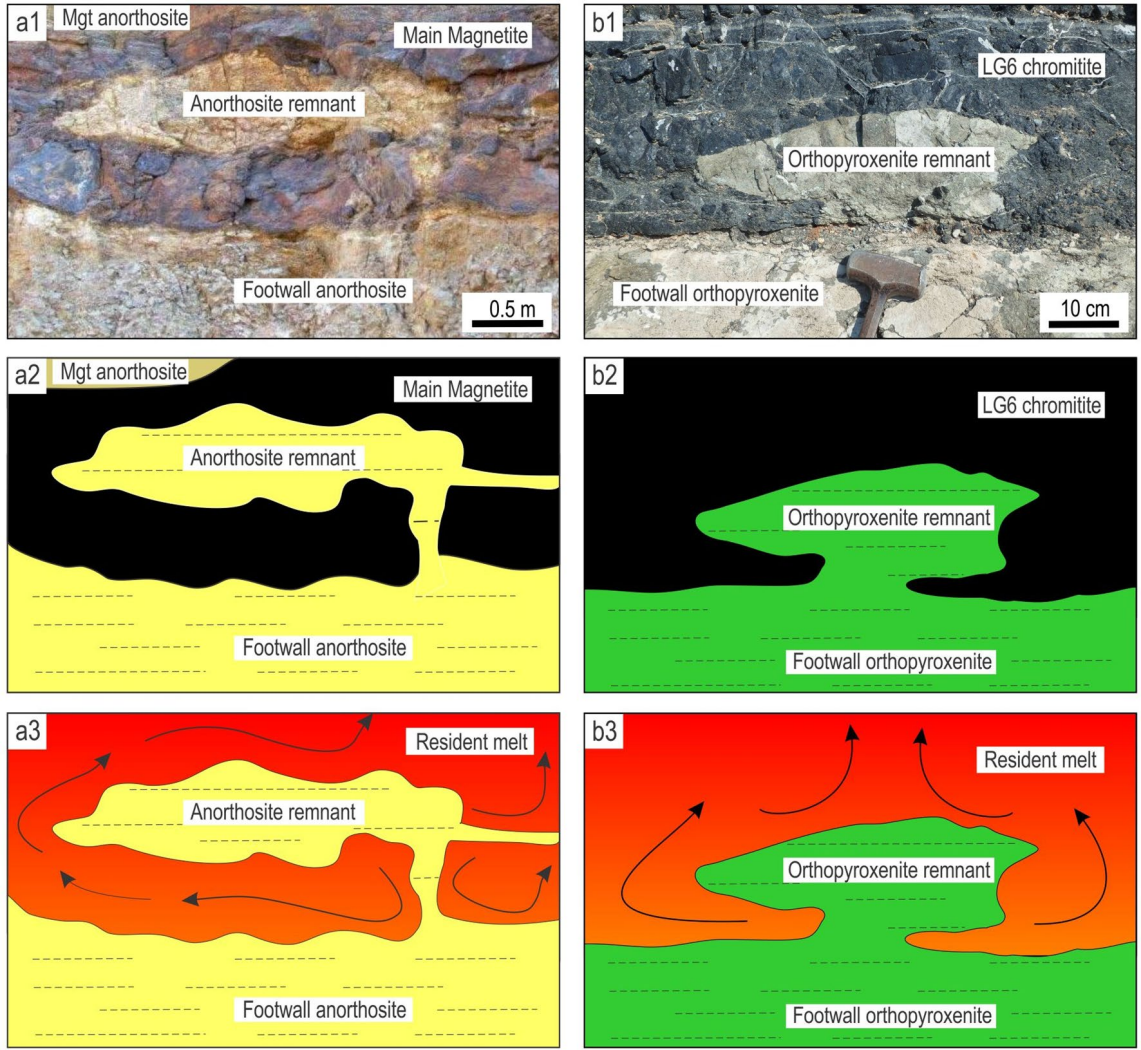

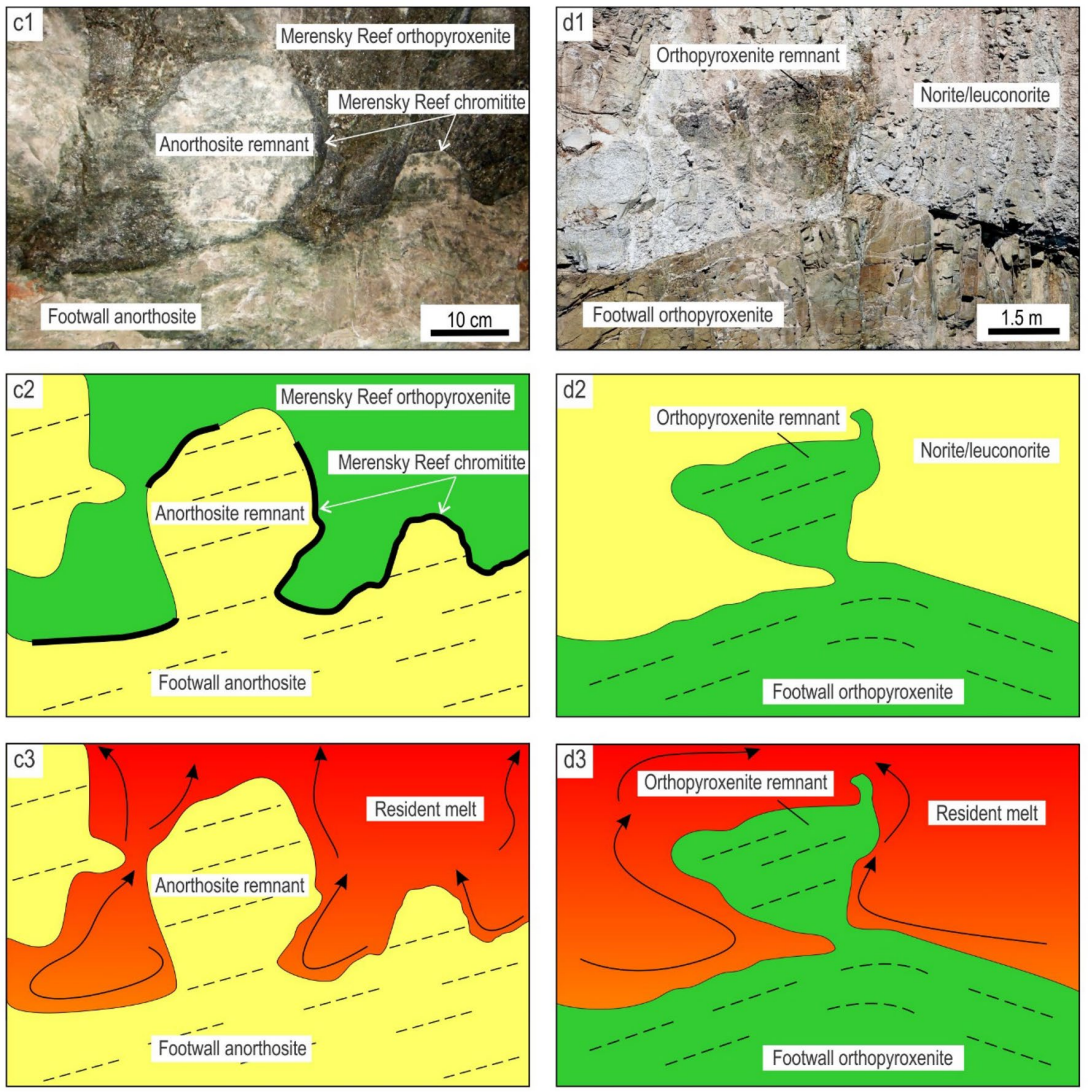

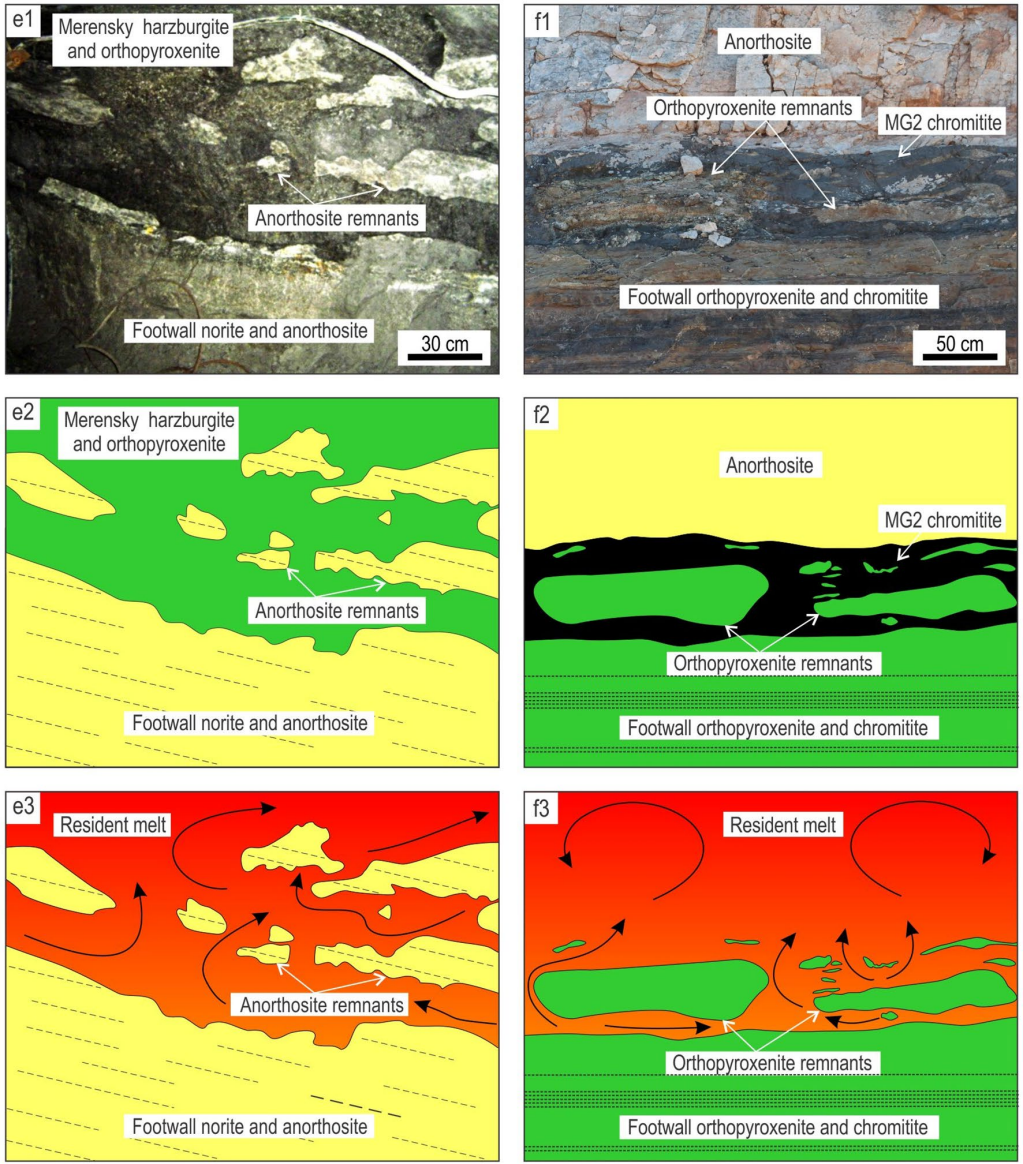
The morphology of the undercut-embayed chamber floor from different localities of the Bushveld Complex, South Africa. **(a1, a2, a3)**, Photo, sketch and reconstruction of an erosional remnant of anorthosite that is hosted by the Main Magnetite Layer and is still attached to the footwall rocks. Vametco Vanadium Mine, Upper Zone of the Western Bushveld Complex. **(b1, b2, b3)**, Photo, sketch and reconstruction of an erosional remnant of orthopyroxenite that is hosted by the LG6 chromitite and is still attached to the footwall rocks. Jagdlust area, Lower Critical Zone of the Eastern Bushveld Complex. The figure is prepared using CorelDRAW (version 18.1.0.690). The morphology of the undercut-embayed chamber floor from different localities of the Bushveld Complex, South Africa. **(c1, c2, c3)**, Photo, sketch and reconstruction of erosional remnants of anorthosite that are hosted by the Merensky Reef orthopyroxenite and are still attached to the footwall rocks. Karee Platinum Mine, Upper Critical Zone of the Western Bushveld Complex. **(d1, d2, d3)**, Photo, sketch and reconstruction of an erosional remnant of orthopyroxenite that is hosted by the overlying norite/anorthosite and is still attached to the footwall rocks. Modikwa Platinum Mine, Upper Critical Zone of the Eastern Bushveld Complex. The figure is prepared using CorelDRAW (version 18.1.0.690). The morphology of the undercut-embayed chamber floor from different localities of the Bushveld Complex, South Africa. **(e1, e2, e3)**, Photo, sketch and reconstruction of in situ erosional remnants of anorthosite that are hosted by the Merensky Reef orthopyroxenite and seemingly not attached to the footwall rocks in this section. Remnants appear to have retained their original positions and orientations. Brakspruit Pothole, Rustenburg Platinum Mine, Upper Critical Zone of the Western Bushveld Complex. **(f1, f2, f3)**, Photo, sketch and reconstruction of in situ erosional remnants of orthopyroxenite that are hosted by the MG2 chromitite and seemingly not attached to the footwall rocks in this section. Remnants appear to have retained their original positions and orientations. Hoggenoeg Chrome Mine, Upper Critical Zone of the Eastern Bushveld Complex. In all the above examples, the morphology of the undercut-embayed floor is attributed to magmatic karstification (i.e. erosion by dissolution) of the chamber floor cumulates by new magma pulses that replenish the chamber. The figure is prepared using CorelDRAW (version 18.1.0.690).

The first postulate that requires attention is that the temporary floor of a magma chamber is planar so that the cumulate pile is always in direct contact with the overlying resident melt^6,8,17,25,26,34,57^. Our data show that this may not be true for magma chambers undergoing repeated replenishments. In this case, the solid floor may be separated from the overlying melt by a few metres thick undercut-embayed floor (Figs. 4, 5). This transitional zone consists of a three-dimensional framework of in situ bodies (i.e. non-transported erosional remnants) that are spatially interconnected with each other and the floor rocks. Revisiting our field observations indicates that the undercut-embayed floor is, in fact, a quite common phenomenon in the Bushveld Complex which has so far escaped our attention. Field evidence for such a floor is abundant at all stratigraphic levels of this complex in the form of erosional remnants that are still partly attached to their footwall (Fig. 5b, c; Fig. 6). More commonly, the erosional remnants occur as isolated fragments that are seemingly ‘suspended’ among host rocks (Figs. 5d, 6). Regrettably, to prove the non-transported nature of these fragments using geochemical mapping does not seem possible because, unlike magnetite, all silicate minerals (e.g. plagioclase, olivine, pyroxenes) and chromite are not chemically sensitive enough to record the evolution and propagation of solidification fronts in magmatic systems in sufficient detail^35^.

Another tenet to be reconsidered is that the resident melt in large basaltic chambers is only cooled by losing heat through the roof rocks once crystallization is underway^18,58,59^. This view stems from a well-known fact that in large layered intrusions heat loss through the chamber floor becomes negligible once a thick cumulate pile has accumulated, insulating the resident melt from the cold country rock below^58,59^. For this reason, after the formation of only ~ 100 m of floor cumulates, in situ nucleation and growth of crystals due to cooling through the chamber floor is considered to be impossible^60^. However, our results suggest otherwise (Fig. 1). Magnetite growth nodes detected in the profile are not randomly distributed but rather tend to be concentrated in the vicinity the largest inclusion X and in a small depression (Fig. 1b). In our previous study^35^_,_ the highest Cr concentration recorded in the Main Magnetite Layer from the Bushveld Complex was also located underneath an anorthositic inclusion. Growth nodes of a similar Cr content are never found directly on the surfaces of the inclusions. Such distribution of the growth nodes can be most logically explained by cooling directly through the floor, while the anorthositic inclusions serve as heat sinks that further aid cooling of the surrounding melt. Where anorthositic inclusions are located close to the floor cooling would be most efficient, explaining why nucleation and the formation of growth nodes are more favourable here. Cooling would also be more efficient within a depression, and magnetite nucleation is more probable here than on a flat surface. The reason why growth nodes with high Cr contents are not located directly on the outer surfaces of inclusions is likely because of their inability to cool the melt as effectively as the thick cumulate pile below. Cooling of melt through the floor in open magma chambers likely becomes important because magmatic karstification excavates the deep and already cold cumulates. It should be noted, however, that cooling through the floor provides only a partial solution to an in situ crystallization mechanism. A major reason why crystals prefer to form on the 3D framework of the undercut-embayed floor is because heterogeneous and self-nucleation on pre-existing crystals is much more favourable due to energy considerations compared to homogeneous nucleation in the main magma body^6,8,61^.

Yet another dogma to be reassessed is that, with progressive cooling, basal solidification fronts in magma chambers invariably propagate unidirectionally upwards until the chamber is completely solidified^5,6,8,13,17,18,25,26,34,57,59,62^. This postulate is invalid for the undercut-embayed floor (Figs. 4, 5) because its three-dimensional framework has multiple cooling surfaces on which crystals may nucleate and grow. As a result, solidification fronts may simultaneously advance in nearly all directions (Fig. 4; Supplementary Information Movie 1) causing them to converge underneath some inclusions (Fig. 3c–e). This realization provides a distinctly different interpretation for Cr reversals that are so common in Bushveld magnetitite layers^36^ (Fig. 3). Because these reversals were found to be laterally discontinuous^36^_,_ they could not be explained by replenishment of the chamber by new Cr-undepleted melts. They also cannot be attributed to convective Cr-undepleted eddies descending from the interior of the magma chamber^36^ because these can hardly penetrate through a three-dimensional framework of the undercut-embayed floor. In contrast, we suggest that these reversals are due to the omnidirectional propagation of solidification fronts from the outer surfaces of anorthositic fragments (Fig. 4). Although such fragments were not reported to be associated with Cr reversals^36^, the reversal in a cryptic zone of magnetite that connects inclusions (Z and Y in Fig. 1b) clearly shows that the fragments may simply not be visible. It is also important to note that not all fragments may be associated with Cr reversals. The reversals may be absent if cooling through the floor is very efficient, so that an upward-propagating solidification front captures the lowermost inclusions before crystals start nucleating and growing on their outer surfaces (Figs. 1, 3a).

### Implications for magma chamber dynamics

A key advantage of the undercut-embayed floor—as a newly recognized petrological realm—is that it provides clear-cut constraints on where, why and how magma crystallizes and differentiates in basaltic magma chambers. In this particular case (i.e. crystallization of magnetitite in the Bushveld Complex), the following solutions to these fundamental questions can be proposed: (1) *where:* magma crystallizes in situ, i.e. directly on all surfaces of three-dimensional framework of the undercut-embayed floor; (2) *why: *in situ crystallization occurs on these surfaces because heterogeneous and self-nucleation on pre-existing crystals of the floor cumulates has the lowest activation energy; in addition, cooling through these surfaces favours crystal nucleation on the undercut-embayed floor. This leaves the final question; (3) *how* do these processes occur within this environment? It is generally accepted that a planar chamber floor is crucial for magmatic differentiation because it allows effective mass transfer between the overlying resident liquid and floor cumulates (e.g. by compaction^63^ or compositional convection^64^ within mushy cumulates). Following this logic, the three-dimensional framework of the undercut-embayed floor would serve as a serious obstacle for magmatic differentiation. In this respect, it is noteworthy that even in extremely confined spaces, such as those below the largest fragment X or where solidification fronts converge (Fig. 1), the mass exchange still occurs extremely effectively to produce a pure magnetite adcumulate. This means that the undercut-embayed floor presents no physical barriers whatsoever for the chemical exchange between the resident melt and the melt in a three-dimensional framework. We can envisage only one mechanism of magma differentiation that is able to operate in such extremely confined spaces. This is compositional convection governed by a gravitational instability of a thin liquid boundary layer around in situ growing magnetite crystals^7,8,35,65^. During magnetite crystallization, such a boundary layer gradually increases in thickness and decreases in density until it obtains sufficient buoyancy to be released upwards into the main magma body, either as a constant stream of melt or as a series of plumes^35^. Fluid dynamic modelling in previous studies have found that such liquid boundary layers may convect after reaching a thickness of just 3 mm^8,35^, ensuring crystal/liquid fractionation occurs in even the narrowest portions of the magmatic karst environment. The mixing of these boundary layers with the overlying resident melt causes its chemical differentiation which is recorded in subsequently forming magnetite nodes and layers^35^.

The last question to address is about the applicability of our findings to other rock-types and other layered intrusions around the world. Magma chamber evolution via in situ crystallization and differentiation in the undercut-embayed floor may be quite common in nature because many large plutonic complexes grow by multiple magma replenishments^8,17,18,25,26,66–69^ which are prone to induce karstification of the floor cumulates^26,46–48^. We predict that many more examples of the undercut-embayed floor will be documented with time in intrusions that show large scale erosional unconformities (e.g. Stillwater^70^, Rum^71,72^, Penikat^67^, Windimurra^68^) such as circular excavations in the chamber floor in which parts of cumulate rocks are missing. However, in between karstification events, the growth of the magma chamber would still likely prevail by a planar solidification front and a pertinent question is whether a physical mechanism of crystallization and differentiation would be different from that inferred from the undercut-embayed floor. At present, we see no obvious reason as to why a transition from the undercut-embayed floor to the planar floor and vice versa may somehow change the magma chamber dynamics (e.g. to replace in situ growth by crystal settling^17^ or crystal mush formation^63,64^. The means by which magma chambers evolve could therefore be similar to other rock types and other layered intrusions as long as the crystallizing minerals have a density higher than that of the melt (e.g. olivine, pyroxene, and chromitite). Since rock types composed of these minerals (e.g. dunite, pyroxenites, and chromitites) are abundant in lower parts of many mafic–ultramafic layered intrusions (e.g. Bushveld Complex, Stillwater Complex, the Great Dyke), the convective-removal of thin boundary layers around in situ growing crystals may be a relatively common process, regardless of whether or not they crystallize in a magmatic karst environment. It is, however, essential that each and every layer be examined individually to determine the means by which the crystals accumulated. For example, while field evidence has been presented for the in situ crystallization of chromitites^34,46,47^, the rocks that contain multiple primocrysts in non-cotectic proportions are still best explained by mechanical deposition of crystal mushes^24,73,74^.

Finally, whether or not in situ crystallization via a planar floor or the undercut-embayed floor will have a different effect on the chemical evolution of a resident melt remains an open question to be addressed in future research. However, as of now it is clear that the undercut-embayed floor is an extremely promising environment for unravelling new fundamentals of igneous petrology.

## Methods

### Chemical analysis and quantification of data

We have analysed an exposure of the bottom magnetitite layer from the Rhovan open pit mine, Western Bushveld Complex (25°34′56.76″ S, 27°34′37.59″ E) on a grid pattern using a portable Niton XL3t XRF analyser. The instrument analyses an area with a diameter of 8 mm. A grid spacing was employed of 4 cm. Each spot of the grid was screened with the portable XRF (pXRF) for about 60 s. We have calibrated the instrument every few hours of its use using its own built-in standards. The following recalculations have been performed to obtain quantitative data from the pXRF. First, we have determined the Cr/V ratio of each analysis. This was done because the surface of magnetitite outcrops was not perfectly planar and it was, therefore, not possible to obtain proper contact with the pXRF for each analysis. For this reason, the actual elemental concentrations in almost all cases are underestimated. Fortunately, if a fair amount of contact between the instrument and rock is maintained, the elemental ratios can be determined accurately. This is evident from the constant V/Ti ratios in our data (V/Ti ratios are also almost-constant for in-house XRF analysis on pure magnetite separates; this is despite the fact that some deviation occurs from the ideal V/Ti ratio if ilmenite grains are incorporated into the analysed spot). We have omitted from the geochemical contour map the spots showing anomalous V/Ti ratios. The recalculated Cr/V ratios are then multiplied by 9.757 to obtain a quantitative Cr concentration in weight %. By doing this, we have got a fit with a linear function f(x) = x between the portable XRF and in-house data and an R-squared correlation coefficient of 0.97 (Supplementary Information Fig. 1). Thereafter we have constructed geochemical contour maps using Surfer Version 9.2.397. The average 2σ analytical uncertainty is 400 ppm and the highest analytical uncertainty is about 1000 ppm. To account for this uncertainty, we have spaced geochemical contours on geochemical contour maps accordingly to ensure they are further apart than the maximum 2σ measured within each region. Supplementary Information Tables 1–3 contains all pXRF data used in our study.

### Velocity of solid particles in a magma

The terminal velocity of spherical particles in a fluid can be calculated with the following equation referred to as Stoke’s Law:

$${\text{v}} = {2}(\rho_{l} -\rho_{{\text{s}}} ){\text{gR}}^{{2}} /{9}\upmu$$

where v is the velocity of a solid particle in the fluid in m/s, $\rho_{l}$ the density of the liquid = 2600 kg/m^3^, $\rho_{s}$the density of the solid particle = 2626 kg/m^3^, g acceleration due to gravity = 9.8 m/s^2^, R the radius of the solid particle = 0.05 m, and µ the dynamic viscosity of the melt = 10^4^ kg/m*s. The solid particle differs in density compared to the liquid by only one percent but may sink by as much as 8.5 m in a week.

### The erosive capability of superheated melt

The formation of the undercut-embayed floor in an open magma chamber requires significant erosion of previously deposited cumulates. A simple way to achieve this is by subjecting the cumulates to superheated melt with which they are in chemical dissequilibrium. Because it is difficult to envision where such a melt will come from within the magma chamber itself, a magma source from outsite the chamber is preferred. Magmas crystallizing at greater depth in some staging chamber are kept at a relatively high liquidus temperature compared to shallower magma bodies because the higher pressure favours crystallization. If a pulse of melt is released upwards from a magma chamber that, say, crystallizes at the Moho seismic discontiuity, such a melt can become superheated by as much as 90 °C relative to the liquidus as it rises adiabatically^54^. In reality, some cooling of the rising melt will inevitably occur in response to resorption of any suspended phenocrysts inherited from the deeper magma reservoir, assimilation of crustal wall rocks, and conductive heat loss to cold crustal rocks. Taking into account these possible cooling mechanisms during ascent, upon arrival into a shallow-level chamber the melt may only be superheated by, say, ~ 15 °C or so. If such a basal flow enters the Bushveld magma chamber, the ratio of melt required to dissolve a certain amount of cumulates (R) can be calculated using the equation below.

$${\text{R}} = (\uprho_{s} {\text{L}}_{{\text{s}}} )/(\uprho_{{\text{l}}} {\text{C}}_{{\text{p}}} \Delta{\text{T}}_{{\text{s}}} );$$

where $\rho_{{\text{s}}}$ = density of the solid = 3.00 g/cm, L_s_—latent heat of the solid = 300 J/g, $\rho_{{l}}$—density melt = 2.6 g/cc, C_p_—heat capacity = 1.34 J/(g °C), $\Delta$T_s_—superheat = 15 °C. Note that in this equation the ($\rho_{{\text{s}}}$L_s_) term is the heat required to dissolve a given volume of solid, whereas the ($\rho_{{l}}$ C_p_$\Delta$T_s_) term is the heat available in the melt for dissolution. Inserting the above values into the equation yields the following results:

$$ {\text{R}} = \left( {{3}.0 \times {3}00} \right)/\left( {{2}.{6} \times {1}.{34} \times {15}} \right) = {17}.{22} $$

Therefore, to dissolve a 5 m thick layer of cumulate rocks would require a basal melt layer that is 86.1 m thick. Such a melt layer would be able to dissolve approximately 5.5 to 10 cm of cumulates per year^55^. Cooling of such a melt layer is expected to be extremely slow (estimated to be approximately 0.013 °C/year for a 1 km thick column of melt^55^), allowing the melt layer to remain superheated for centuries. The above calculations are sensitive, however, to input parameters. For instance, we can substantially decrease the magma column that is needed to melt the rocks by increasing the degree of magma superheating and vice versa. In a similar way, we can double the rate of the dissolution process by taking the rate of dissolution at the higher end. Thus, only slight superheating of the melt relative to its liquidus temperature may allow erosion of a substantial amount of footwall rocks through dissolution/melting. Such a superheated melt has enough heat to accomplish the required dissolution/melting. Importantly, during this process the melt will not crystallize so that it will be in a continuous direct contact with the floor rocks.

## Supplementary Information

Supplementary Video Legend.

Supplementary Video 1.

Supplementary Figure 1.

Supplementary Table 1.

Supplementary Table 2.

Supplementary Table 3.

## Data Availability

All data generated or analyzed during this study are included in this published article (and its Supplementary Materials Files).
