# Supplementary material for: Magmatic karst reveals dynamics of crystallization and differentiation in basaltic magma chambers
Source: Sci Rep. 2021 Apr 1;11:7341. doi: 10.1038/s41598-021-86724-y (PMC8016925; doi:10.1038/s41598-021-86724-y)
Supplement: Supplementary file 1 — Supplementary Video Legend. [file 41598_2021_86724_MOESM1_ESM.docx]

**Supplementary Movie 1*.* Propagation of a solidification front of massive magnetitite in the undercut-embayed chamber floor.** Magnetitite starts nucleating and growing in localities where cooling is most significant (directly on the floor in the vicinity of inclusions and a depression towards the left) to produce growth nodes. This is followed by rapid lateral growth until the floor is mostly covered. Thereafter, magnetite starts growing on the outer surfaces of inclusions and from the floor upwards. Where solidification fronts converge (a left side of the image), Sandwich Horizons emerges.
