# Supplementary material for: Magmatic karst reveals dynamics of crystallization and differentiation in basaltic magma chambers
Source: Sci Rep. 2021 Apr 1;11:7341. doi: 10.1038/s41598-021-86724-y (PMC8016925; doi:10.1038/s41598-021-86724-y)
Supplement: Supplementary file 3 — Supplementary Figure 1. [file 41598_2021_86724_MOESM3_ESM.docx]

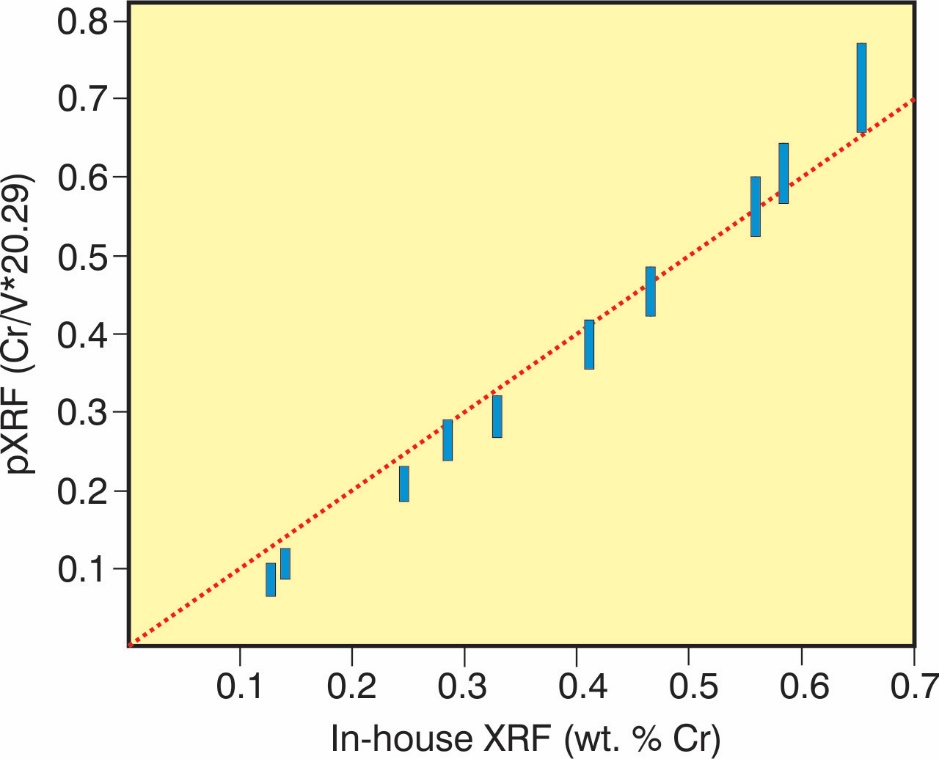


Supplementary information Fig. 1. Calibration curve for the portable XRF (pXRF) against in-house XRF data. Hand samples collected from the bottom seam shown in Figure 1a were analysed via the pXRF. In-house XRF data were obtained from pure magnetite separates from the same samples. The vertical spacing of the blue bars represents the 2Θ analytical uncertainty of the pXRF analysis. In-house XRF data were obtained from pure magnetite separates.
